# Post-traumatic growth during the COVID-19 pandemic in carers of children in Portugal and the UK: cross-sectional online survey

**DOI:** 10.1192/bjo.2021.1

**Published:** 2021-01-20

**Authors:** Paul Stallard, Ana Isabel Pereira, Luísa Barros

**Affiliations:** Department of Health, University of Bath, UK; Psychology Research Centre, Faculty of Psychology, University of Lisbon, Portugal; Psychology Research Centre, Faculty of Psychology, University of Lisbon, Portugal

**Keywords:** Carers, COVID-19, post-traumatic growth, cross sectional, well-being

## Abstract

**Background:**

Although the negative effects of the COVID-19 pandemic on mental health have attracted interest, little attention has focused on its positive effects and possible post-traumatic growth.

**Aims:**

To assess anxiety, well-being and post-traumatic growth in carers of children aged 6–16 years in Portugal and the UK.

**Method:**

A cross-sectional online survey of volunteers conducted at the peak of the first wave of COVID-19 during lockdown (1 May to 27 June 2020).

**Results:**

A total of 385 caregivers (Portuguese, *n* = 185; UK, *n* = 200), predominantly mothers (*n* = 341, 88.6%), completed the survey. The majority were working exclusively from home (*n* = 271, 70.4%), almost half reported a reduction in income (*n* = 174, 45.2%), most children were home taught (*n* = 358, 93%), and 75 (19.5%) identified a family member with suspected or confirmed COVID-19 infection. In total, 341 caregivers (88.6%) identified positives arising from COVID-19, most commonly related to the post-traumatic growth domains of improved relationships, a greater appreciation of life, discovering and embracing new possibilities, and positive spiritual change. A comparison of those who did (*n* = 341) and did not (*n* = 34) report any positives found a significant difference in well-being scores (*t*_373_ = 2.24, *P* = 0.025) but not in anxiety scores (*t*_373_ = 0.75, *P* = 0.45).

**Conclusions:**

Despite experiencing considerable adversity, examples of post-traumatic growth during the lockdown were common. Although the voluntary online nature of our survey is a limitation, our findings suggest that further research exploring post-traumatic growth following pandemics is warranted.

On 11 March 2020 the World Health Organization (WHO) declared the outbreak of COVID-19 to be a global pandemic.^1^ By 10 November 2020 there were 50 266 033 confirmed cases of COVID-19 worldwide, with 1 254 567 deaths reported to the WHO.^2^ The immediate medical emergency and subsequent attempts to contain the spread of the virus resulted in many countries adopting local or national lockdowns. The primary focus was on reducing infection and death rates, but the effects of lockdown, home working and social containment on mental health were soon identified.^3,4^ However, much of the published data have been obtained from cross-sectional surveys of self-completed questionnaires undertaken at different times during the pandemic, and there is an absence of well-designed, repeated longitudinal surveys.^5^ Preliminary findings from these surveys are variable, but in general they have identified increased rates of anxiety,^6,7^ depression^8^ and post-traumatic symptoms.^9,10^

In terms of children and young people, the medical consequences of COVID-19 infection are less severe, with hospitalisation and mortality rates tending to be low.^11^ However, the psychological effects of the pandemic and the public health response have created significant pressures which may place children and young people at particular risk.^5,11^ Educationally, children have had their education disrupted through school closures, online education and uncertainty about examination arrangements.^11^ An unintended consequence of quarantine and enforced isolation is loneliness, a factor that is particularly problematic for children and young people, who tend to be heavily dependent on their peers.^12^ In addition, children may be at increased risk of witnessing domestic violence or directly experiencing abuse as their carers struggle to cope with the cumulative pressures of the pandemic.^13^

Carers have a key role in protecting and buffering their children from the effects of COVID-19 and lockdown. In addition to their own stresses, arising from uncertainty, disrupted work, reduced personal finances, less social support and health concerns, they also assume additional responsibilities for maintaining family life and for educating their children.^14^ It is therefore not surprising that cross-sectional studies have detailed the adverse effects of COVID-19 on the mental health of carers.^14–16^

Although the negative sequelae of COVID-19 on mental health have been detailed, little is known about the potential positive psychological effects of the pandemic and lockdown and whether this might trigger post-traumatic growth.^17,18^ Post-traumatic growth is a positive, adaptative process where traumatic experiences are re-evaluated, and a new and positive life narrative developed.^18^ Typically, post-traumatic growth follows a disruptive physical or psychological trauma that challenges an individual's perspectives, values and roles. Growth arises from the way the event is processed, not from the event itself, and leads individuals to recognise their vulnerability and what they can and cannot control, and to reassess their personal priorities. This leads to the revision and development of new psychological and philosophical beliefs and stimulates growth across three domains: self-perception, interpersonal relationships and life philosophy.^19^ This can result in changes in five main areas: improvements in relating to others, greater personal strength, positive spiritual change, a greater appreciation of life, and discovering and embracing new possibilities.^19^

There is evidence that people of all ages who have experienced various types of trauma can identify positive ways in which their lives have changed and that these are related to improved mental health and well-being.^20^ Indeed, reviews have identified that up to 50% of those who have experienced a trauma experience some degree of post-traumatic growth.^21^ Given the reach of the COVID-19 pandemic and the current focus on negative mental health sequelae, exploration of potential post-traumatic growth and whether this might maintain and promote well-being might be an alternative paradigm to investigate.^17^

Our study aimed to investigate the positive psychological effects of the COVID-19 pandemic and lockdown and post-traumatic growth in a particularly vulnerable group, carers of children, in Portugal and the UK.

## Method

### Design

This project is part of a European study investigating the association between cumulative risk factors and child and parent mental health during the COVID-19 pandemic. The study approval and set-up process resulted in countries starting recruitment at different times. The data presented here are from the UK and Portugal, the two countries who began recruitment during the peak of the first wave of the pandemic. Data were collected during lockdown between 1 May and 27 June 2020, following the period with the highest incidence of new cases and mortality in each country.

The study was cross-sectional and carers of children aged 6–16 years in Portugal and the UK were invited to participate in an online survey evaluating how the carers were coping with COVID-19 and lockdown. The online survey and data collection tool were developed using the Qualtrics online survey tool hosted by the Faculty of Psychology, University of Lisbon. The study was approved by the ethics committees at the Universities of Lisbon (Portugal) and Bath (UK). Recruitment was via social media, email, institutional advertising and websites, with consent provided online. Before giving consent to participate, caregivers received all the information concerning the study's objectives, the voluntary nature of their participation and the anonymity of the data collected.

### Measures

#### Demographic information

Information was collected about carer demographics (age, gender, education and work status), family type (nuclear family), family size (number of children), effects of the COVID-19 pandemic (child home schooled, parents working remotely, reduction in income) and experience of COVID-19 (family or close friend contracted COVID-19).

#### Caregiver anxiety

To measure caregiver anxiety, we used the Generalised Anxiety Disorder 7-Item Scale (GAD-7).^22^ The GAD-7 is composed of 7 items (e.g. not being able to stop or control worrying). Carers rated each item on a four-point Likert frequency scale, ranging from not at all to nearly every day over the period of the preceding 2 weeks. Higher scores indicate higher levels of anxiety, with a cut-off score of ≥10 representing moderate levels of anxiety suggesting further assessment. The alpha for the current sample was 0.90.

#### Caregiver well-being

The Warwick–Edinburgh Mental Wellbeing Scale (WEMWBS)^23^ was used to measure caregiver well-being. The WEMWBS consists of 14 items that assess positive affect (e.g. ‘I've been feeling confident’) satisfying interpersonal relationships (e.g. ‘I've been feeling close to other people’) and positive functioning (e.g. ‘I've been thinking clearly’). Carers rated each item on a five-point Likert scale (from none of the time to all of the time) over the time frame of the preceding 2 weeks. The total score ranges from 14 to 70, with higher scores indicating higher levels of well-being. A cut-off of ≤40 has been found to indicate a high risk of depression.^23^ The alpha for the current study was 0.92.

#### Post-traumatic growth

This was assessed via a single open question: ‘Do you think there are any positives to come out of this pandemic and the social distancing restrictions?’.

### Data analysis

Quantitative data were summarised with descriptive statistics. Student's *t*-tests explored differences between scores on standardised questionnaires, with non-parametric χ^2^-tests examining categorical data. Qualitative data were subject to thematic analysis.^24^ Responses were transcribed verbatim as the researchers familiarised themselves with the data. The responses were then divided into short segments, which were coded according to complementary deductive and inductive processes. There was no *a priori* coding framework, and the researchers were masked to the specific domains of post-traumatic growth during coding. An initial reading of all codes allowed a first definition of emerging themes. Themes were reviewed by two of the authors (A.I.P. and L.B.) and were refined through multiple iterations until final agreement and data saturation were reached. A Portuguese researcher (A.I.P.) checked the classification for codes produced by Portuguese participants and the English researcher (P.S.) verified those for British participants. Whenever there was a disagreement, a third researcher would resolve it. After codes were finalised, descriptive statistics were calculated to evaluate the absolute and relative frequency of each theme using each participant as a unit of count. A χ^2^-test of independence was performed to examine the relation between the country (Portugal and UK) and each theme.

Finally, Spearman correlations evaluated the relationship between the perception of positives and carers’ mental health.

## Results

### Demographics

The participants (Portuguese, *n* = 185; UK, *n* = 200) were predominantly mothers (*n* = 341, 88.6%), aged 40–49 years (*n* = 226, 58.7%), living in an intact nuclear family (*n* = 307, 79.7%), with one or two children (*n* = 333, 86.5%) (Table 1).

**Table 1:** Demographics and COVID-19 experience of carers of children in Portugal (*n* = 185) and the UK (*n* = 200)

Variable	Portugal, *n* (%)	UK, *n* (%)	χ^2^
Respondents			0.102
Mother	158 (85.4)	183 (91.5)	
Father	25 (13.5)	14 (35.9)	
Other	2 (1.1)	3 (1.5)	
Respondents’ age, years			3.59
<30	0 (0)	3 (1.5)	
30–39	55 (29.7)	56 (28.0)	
40–49	111 (60.0)	115 (57.5)	
>50	19 (10.3)	26 (13.0)	
Respondents’ education			17.31***
No college degree	20 (10.8)	44 (22.0)	
University graduate	65 (35.1)	87 (43.5)	
University postgraduate	100 (54.1)	69 (34.5)	
Respondents’ work status			111.23***
Full-time work	164 (88.6)	79 (39.5)	
Part-time work	6 (3.2)	96 (48.0)	
Other	15 (8.1)	25 (12.5)	
Intact nuclear family	142 (76.8)	165 (82.5)	1.96
Children in the household			11.55**
1	78 (42.2)	52 (26.0)	
2	87 (47.0)	116 (58.0)	
3 or more	20 (10.8)	32 (16.0)	
Child in home schooling	181(97.8)	177 (88.5)	12.85***
Adults in the family exclusively working remotely			4.97
0	48 (25.9)	66 (33.0)	
1	88 (47.6)	73 (36.5)	
2 or more	49 (26.5)	61 (30.5)	
Income reduction			17.51***
None	106 (57.3)	105 (52.5)	
Less than 30%	36 (19.5)	72 (36.0)	
More than 30%	43 (23.2)	23 (11.5)	
Anyone in the family or close friend contracted COVID-19			41.20***
No	173 (93.5)	137 (68.5)	
Infected/suspected	11 (5.9)	63 (31.5)	
Died	1 (0.5)	0 (0.0)	

*P < 0.05; **P < 0.01; ***P < 0.001.

The majority were working exclusively from home (*n* = 271, 70.4%). Almost half reported a reduction in income (*n* = 174, 45.2%), with most children receiving remote schooling (*n* = 358, 93%). In total, 75 (19.5%) carers reported a family member with suspected or confirmed COVID-19 infection, with this being more likely in the UK sample. Portuguese carers were more likely to have one child residing in their household, to be in full-time employment, to have a postgraduate degree and to suffer a reduction of income of more than 30%.

### Anxiety and well-being

Well-being scores (on the WEMWBS) were higher for Portuguese (mean 47.94, s.d. = 7.75) than for UK (mean 45.51, s.d. = 9.05) carers (*t*_383_ = 2.81, *P* = .005), with 99 carers (25.7%) scoring ≤40, suggesting a risk of depression. There was a between-country difference on the WEMWBS (χ^2^(1) = 8.61, *P* = 0.003), with more carers in the UK (*n* = 64) falling below the threshold than in Portugal (*n* = 35).

Anxiety scores (on the GAD-7) were also slightly higher in Portuguese (mean 6.90, s.d. = 4.54) than UK (mean 5.98, s.d. = 4.82) carers but this difference was marginal (*t*_383_ = 1.93, *P* = 0.054). A total of 83 carers (21.6%) scored ≥10 on the GAD-7, suggesting moderate anxiety, but there was no difference between countries (Portugal, *n* = 43; UK, *n* = 40; χ^2^(1) = 0.59, *P* = 0.44). These results are in line with previous surveys, in which 23% of primary care patients scored ≥10.^22^

### Post-traumatic growth

In total, 341 respondents (88.6%) felt that there were positives to emerge from COVID-19 and lockdown, with 309 (80.3%) providing examples. More positives were identified in the UK than in Portugal (χ^2^(1) = 5.78, *P* = 0.16). Thematic analysis identified ten categories (Table 2), which were related to four of the five domains of post-traumatic growth. The most commonly cited category related to the development of closer and more meaningful relationships. This was reflected by growth in family relationships (*n* = 162, 47.5%), described by one participant as ‘closer relationships and a better understanding of each other’. Comments coded under this theme highlighted how carers reported spending more time together as a family, had more involvement in their children's lives, and felt closer and more connected to other family members. A second domain of post-traumatic growth, a greater appreciation of life, was reflected through the reassessment of personal values and priorities (*n* = 76, 22.3%) and the opportunity to ‘reconsider what's really important in life’. This involved increased appreciation and gratitude for a simpler life, with opportunities for ‘reconnection with small pleasures’ with less consumerism and reliance on material things. Another category reflecting this domain was the adoption of a healthier lifestyle (*n* = 75, 22.0%) in which ‘life has slowed down’, resulting in less stress and ‘an opportunity to enjoy the garden and the quiet of the day’. The post-traumatic growth domain of spiritual growth involves a greater engagement with fundamental existential issues. Examples were provided in the category of social growth (*n* = 53, 15.5%), such as ‘people were helping each other more’ and the development of a ‘stronger sense of community’. There was a ‘greater appreciation of others’ (e.g. healthcare workers) and an ‘acknowledgement of inequalities’. Others identified wider environmental benefits (*n* = 52, 15.2%) through ‘less car use’ creating less air pollution, which was ‘better for the environment’. The fourth domain of post-traumatic growth, discovering and embracing new possibilities, was reflected in comments about changes in working practice (*n* = 38, 11.1%), involving positive ‘changes in attitudes to home working’ and the adoption of a ‘better work–family balance’. It was also reflected through opportunities to learn or develop new skills (*n* = 22, 6.5%), particularly ‘acquiring new technology-related competencies’. Respondents highlighted these as particularly important as they increasingly relied on technology for work, educating their children and socialising. Others described the positive opportunity to home educate their children (*n* = 10, 2.9%), with one carer reporting that ‘I have always wanted to home school but cannot afford to […] this has been a wonderful experience’. Finally, there were a few examples that were more descriptive rather than indicative of post-traumatic growth. A few mentioned disease control (*n* = 17, 5.0%) and the ‘benefits of the quarantine and social distancing to avoid or control the spread of the coronavirus infection’. Finally, a few commented on financial benefits (*n* = 6, 1.8%) where ‘staying home helped to save money’.

**Table 2:** Post-traumatic growth themes identified by carers of children in Portugal (*n* = 157) and the UK (*n* = 184)

Themes	Portugal, *n* (%)	UK, *n* (%)	χ^2^
Relating to others			
Opportunity to be with family and improve relations, e.g. closer relationships and a better understanding of each other	65 (41.4)	97 (52.7)	7.10**
Greater appreciation of life			
Opportunity to assess personal values, e.g. reconsider what is really important in life, reconnection with small pleasures	35 (22.3)	41 (22.3)	0.15
Opportunity related to a healthier lifestyle, e.g. life slowing down, staying home, enjoying the garden and the quiet of the day	16 (10.2)	59 (32.1)	26.71***
Discovering and embracing new possibilities			
Opportunity to improve competencies, e.g. learning new skills, acquiring new technology-related competencies	12 (7.6)	10 (5.4)	0.40
Opportunity for work changes, e.g. change in attitudes towards home working, better work–family balance	17 (10.8)	21 (11.4)	0.18
Opportunities for changes in the child's schooling and learning, e.g. assuming responsibility for the child's education	3 (1.9)	7 (3.8)	1.33
Spiritual growth			
Opportunity for social changes, e.g. people helping each other more, acknowledgement of inequalities, a stronger sense of community	16 (10.2)	37 (20.1)	7.85**
Opportunity for environmental changes, e.g. less car use so better for the environment	13 (8.3)	39 (21.2)	12.80***
Other			
Pandemics and disease control, e.g. benefits of the quarantine and social distancing to avoid or control the spread of the coronavirus infection	9 (5.7)	8 (4.3)	0.17
Financial benefits, e.g. staying home helped to save money	0 (0)	6 (3.3)	5.63

*P < 0.05; **P < 0.01; ***P < 0.001.

### Post-traumatic growth, well-being and anxiety

The number of positives identified was correlated with less carer-reported anxiety (*r* = –0.15, *P* = 0.003) and improved well-being (*r* = 0.17, *P* = 0.001). A comparison of those who did (*n* = 341) and did not (*n* = 34) report any positives found a significant difference in well-being scores (*t*_373_ = 2.24, *P* = 0.025) but not in anxiety scores (*t*_373_ = 0.75, *P* = 0.45). Those who failed to identify any positives had lower well-being scores (mean 43.56, s.d. = 8.61) than those who identified positives (mean 47.00, s.d. = 8.51).

## Discussion

Our survey was undertaken during lockdown as the death rate from COVID-19 was peaking. Carers reported severe disruption to work and their children's education; almost half reported a reduced income, and one in five reported suspected or actual family COVID-19 infection. Despite this adversity, the majority identified positives arising from the pandemic and lockdown, with most examples suggesting evidence of post-traumatic growth reflected in changes in behaviour or cognition. These related to the post-traumatic growth domains of relating to others, a greater appreciation of life, discovering and embracing new possibilities and positive spiritual change. We found no clear evidence of the fifth domain, relating to increased personal strength.

The rates of post-traumatic growth reported here are higher (88.6%) than those reported in reviews looking at growth following a range of traumas (52.6%)^21^ or recent studies of nurses during the COVID-19 pandemic (39.3%).^25^ Possible explanations for this difference could be threefold. First, this may reflect our reliance on a single open-ended question to assess post-traumatic growth rather than an established multi-question inventory such as the Post-Traumatic Growth Inventory.^19^ Second, our criteria for identifying post-traumatic growth only required the identification of a single event. The criteria for determining possible post-traumatic growth using standardised questionnaires are typically based on cumulative scores where a number of items must be identified. Finally, our open-ended question may have facilitated post-traumatic growth by prompting a positive mindset that encouraged respondents to actively seek and acknowledge positive developments. Our question may therefore have encouraged respondents to identify and cognitively process events in a more positive way, thereby explaining why rates of post-traumatic growth were so high. Nonetheless, the examples provided in our survey were not abstract or idealistic *post hoc* reconstructions of change but reflected actual changes in behaviour typically as a response to the physical limitations imposed by the lockdown. Further studies involving follow-ups are required to substantiate these findings and to determine whether these changes become established and persist once lockdown is eased.

### Strengths and limitations

Our study is one of the first to explore benefit finding and potential post-traumatic growth arising from the COVID-19 pandemic and lockdowns. The prospective nature of our study is a strength, with data collected in real time during the peak of the pandemic. However, our study does have a number of limitations that need to be acknowledged. First, we report on a small, convenience sample of volunteers and our cohort is therefore not representative of the general population. Indeed, our sample primarily comprised highly educated mothers living within an intact nuclear family. Second, the cross-sectional nature of our design limits the conclusions that can be drawn. Although we can describe our data at a single point in time, we are unable to draw any conclusions about the nature of relationships or how these might change over time. Third, benefits and potential post-traumatic growth were assessed by a single item, not by a validated questionnaire of post-traumatic growth. Although this limitation is acknowledged, our qualitative approach did allow respondents to disclose their experiences and thoughts without constraint. Similarly, given the online delivery of the survey, respondents could easily have skipped this question if they felt it was not relevant or applicable. Given that our qualitative analysis identified core categories commonly reflected in the post-traumatic growth literature adds to the strength and validity of our findings.^19^

### Implications

The positives that participants identified as arising from the COVID-19 pandemic were associated with less anxiety and improved well-being, raising the possibility that post-traumatic growth might mitigate some of the adverse psychological effects of the pandemic. However, our findings need to be interpreted with caution, since our study has a number of methodological limitations. Further research exploring possible post-traumatic growth following the pandemic and the potential positive effects on mental health is warranted.

## Data Availability

The data that support the findings of this study are available on request from the corresponding author. The data are not publicly available because they contain information that could compromise the privacy of research participants.
